# State-of-the-art neonatal cerebral ultrasound: technique and reporting

**DOI:** 10.1038/s41390-020-0776-y

**Published:** 2020-03-26

**Authors:** Jeroen Dudink, Sylke Jeanne Steggerda, Sandra Horsch, eurUS.brain group, Thais Agut, Ana Alarcon, Roberta Arena, Marco Bartocci, Mayka Bravo, Fernando Cabañas, Nuria Carreras, Olivier Claris, Jeroen Dudink, Monica Fumagalli, Paul Govaert, Sandra Horsch, Alessandro Parodi, Adelina Pellicer, Luca Ramenghi, Charles C. Roehr, Sylke Steggerda, Eva Valverde

**Affiliations:** 1 Department of Neonatology, University Medical Center Utrecht, Wilhelmina Children’s Hospital, Utrecht, The Netherlands; 2 Department of Neonatology, Leiden University Medical Center, Leiden, The Netherlands; 3 Department of Neonatology, Helios Klinikum Berlin Buch, Berlin, Germany; 4 Department Clinical Science Intervention and Technology (CLINTEC), Karolinska Institutet, Stockholm, Sweden; 5 Department of Neonatology, Institut de Recerca Pediàtrica, Hospital Sant Joan de Déu, Barcelona, Spain; 6 Catholic University of the Sacred Heart, A. Gemelli Hospital, Rome, Italy; 7 Department of Women’s and Children’s Health, Karolinska University Hospital, Karolinska Insitute, Stockholm, Sweden; 8 Department of Neonatology, La Paz University Hospital, Madrid, Spain; 9 Department of Neonatology, Quironsalud Madrid University Hospital and Biomedical Research Foundation, La Paz University Hospital Madrid, Madrid, Spain; 10 Service de néonatologie et de réanimation néonatale, Hospices Civils de Lyon, Université Claude Bernard Lyon, Lyon, France; 11 Department of Clinical Sciences and Community Health, University of Milan, Milan, Italy; 12 Fondazione IRCCS Ca’ Granda Ospedale Maggiore Policlinico NICU, Milan, Italy; 13 Department of Neonatology, Erasmus Medical Center University, Sophia Children’s Hospital, Rotterdam, The Netherlands; 14 Department of Neonatology, ZNA Middelheim, Antwerp, Belgium; 15 Department of Rehabilitation and Physical Therapy, Gent University Hospital, Gent, Belgium; 16 Neonatal Intensive Care Unit, Istituto Giannina Gaslini, Via Gaslini 5, 16148 Genoa, Italy; 17 Department of Paediatrics, Medical Sciences Division, Newborn Services, University of Oxford, Oxford, UK

## Abstract

In the past three decades, cerebral ultrasound (CUS) has become a trusted technique to study the neonatal brain. It is a relatively cheap, non-invasive, bedside neuroimaging method available in nearly every hospital. Traditionally, CUS was used to detect major abnormalities, such as intraventricular hemorrhage (IVH), periventricular hemorrhagic infarction, post-hemorrhagic ventricular dilatation, and (cystic) periventricular leukomalacia (cPVL). The use of different acoustic windows, such as the mastoid and posterior fontanel, and ongoing technological developments, allows for recognizing other lesion patterns (e.g., cerebellar hemorrhage, perforator stroke, developmental venous anomaly). The CUS technique is still being improved with the use of higher transducer frequencies (7.5–18 MHz), 3D applications, advances in vascular imaging (e.g. ultrafast plane wave imaging), and improved B-mode image processing. Nevertheless, the helpfulness of CUS still highly depends on observer skills, knowledge, and experience. In this special article, we discuss how to perform a dedicated state-of-the-art neonatal CUS, and we provide suggestions for structured reporting and quality assessment.

## Introduction

Cerebral ultrasound (CUS) is still the first-line neuroimaging modality to study the neonatal brain. It is less expensive and burdensome than magnetic resonance imaging (MRI), which requires patient transport and sometimes sedation. CUS can be performed bedside with acceptable disturbance to the infant. The procedure is radiation-free and can be initiated directly after birth, providing quick images in real time. Serial imaging can provide valuable information about the timing and evolution of brain lesions during the course of brain maturation.^1,2^ Since the introduction of CUS in neonatal care in the late 1970s,^3^ its quality has dramatically improved. Modern US systems provide increasingly higher resolution and faster image processing. In the past, CUS exams were mostly performed to depict the ventricular system and to diagnose intraventricular hemorrhage (IVH) and periventricular cysts.^3–6^ Currently, CUS provides more details and a trained observer can detect most neonatal hemorrhagic and ischemic brain lesions, major congenital anomalies, and maturational changes in both preterm and term infants.^7–11^ Early identification of infants with brain injury and thus at risk of neurodevelopmental impairment is now thought to benefit the individual infant, because appropriate early referrals can be made allowing to initiate interventions aimed at improving neurological outcome. The use of high-frequency transducers further improved visualization of both superficial and deep areas of the brain.^7–12^ Additional acoustic windows: posterior fontanel, mastoid fontanel, temporal window, and foramen magnum, extended visualization to areas less accessible via the most commonly used anterior fontanel (AF), resulting in a more reliable detection of abnormalities.^13–20^ Neonatal CUS examinations now routinely include Doppler sonography, with which the patency of both arteries and veins, flow velocities, and variant anatomy can be assessed.^21^ Doppler sonography is highly specific to rule out sinovenous thrombosis at vulnerable vessels.^22,23^ With modern Doppler techniques, we can also quantify low flow velocities in smaller vessels.^24^ Despite the fact that MRI has become more widely available and in some conditions is still the gold standard for diagnosing various neonatal brain injuries, CUS truly deserves a place in brain imaging for its options and accuracy. This special article aims to provide a toolkit for structured neonatal CUS imaging, reporting, and quality assessment.

## Indications for CUS

Postnatal screening with CUS is indicated for all newborns at risk of (or suspected of) brain injury. Three main categories of neonatal brain injury are distinguished according to when it occurred (antenatal, perinatal, and postnatal; Table 1).

**Table 1:** Risk factors and clinical signs of neonatal brain injury.

Antenatal	Abnormal fetal neuroimaging, twin-related problems, intrauterine intervention, antenatal infection (with CMV, Toxoplasmosis, Herpes, Rubella, Syphilis, or other neurotrophic pathogens), fetomaternal transfusion, maternal drug abuse, maternal accidents, and severe illness
Perinatal	Need for prolonged resuscitation, hypoxic–ischemic encephalopathy, prematurity, very low birth weight, small for gestational age, microcephaly and macrocephaly, suspected (genetic) syndrome
Postnatal	Seizures, central apnea, encephalopathy, sepsis/meningitis/encephalitis, unexplained clinical deterioration, unexplained drop in hemoglobin level, symptomatic hypoglycemia, inborn errors of metabolism, preterm kernicterus, abnormal movements or tone, severe arterial hypotension or hypertension, congenital heart disease, need for surgery, or extracorporeal membrane oxygenation

## Timing of CUS examination

In some situations, a single postnatal CUS scan suffices to either confirm or rule out a suspected abnormality. In other conditions, however, such as premature birth, neonatal encephalopathy, or perinatal arterial ischemic stroke, serial examination is mandatory to detect the full spectrum of lesional change.^2^

### Prematurity

In preterm infants born before 28 weeks of gestation or with or a birth weight <1000 g, serial CUS is recommended on days 1, 3, 7, 14, 21, and 28 and then every other week until term-equivalent age because of a high risk of brain injury. In stable preterm infants born after 28 weeks of gestation, the frequency of serial CUS can be limited to days 1, 3, 7, 14, 28, at 6 weeks, and at term-equivalent age.^25^ Additional scans outside suggested schedules should be performed whenever clinically indicated. The first CUS after admission serves to rule out antenatal brain injury and congenital malformation.^25^ The scans during the first week of life aim to detect germinal matrix–IVH, periventricular hemorrhagic infarction, and cerebellar hemorrhage.^26–30^ In at least 50% of the affected infants, the onset of germinal matrix–IVH is on the first day of life, and by 72 h approximately 90% of the lesions are identified.^30,31^ The scans between weeks 2 and 6 help identify post-hemorrhagic ventricular dilatation, white matter injury, focal arterial infarction, sequelae of brain infection, and rare cases of late IVH. Cystic white matter injury (also known as cystic periventricular leukomalacia) may become apparent within 14 days after the insult, although occasionally small cysts may develop up to 6 weeks after birth.^32^ Therefore, carefully performed serial scanning with a high-resolution probe (≥7.5 MHz) after 2 weeks of life is essential to detect all cases of white matter injury. Scanning at term-equivalent age permits assessing how the brain developed and permits identifying permanent residuals of white and gray matter injury.^33^ The value of this late scan for the prediction of outcome of extremely preterm infants is increasingly recognized,^34,35^ the more so at term-equivalent age as its predictive value is comparable to that of conventional MRI.

### Hypoxic–ischemic encephalopathy

**Fig. 1:**
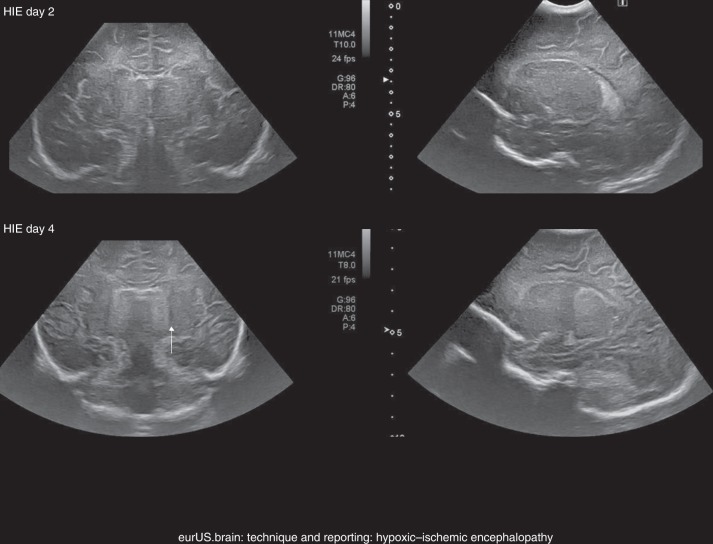
Technique and reporting: hypoxic–ischemic encephalopathy. Intrapartum asphyxia. Term infant, born at 41 weeks’ gestation with asphyxia and hypoxic–ischemic encephalopathy, treated with hypothermia. **a**, **b** Ultrasound on admission showing subtle increased echogenicity of the thalami on the coronal (**a**) but not on the sagittal (**b**) images. **c**, **d** Three days later, there is clearly abnormal increased echogenicity of the thalami in both planes (arrows), which are separated from more mildly echogenic basal ganglia by a band of low echogenicity, representing the posterior limb of the internal capsule (arrowhead).

CUS permits detecting brain injury related to a perinatal hypoxic–ischemic insult.^36^ Brain swelling and impaired perfusion are often seen at an early stage but the hyperechogenicity that is found typically in the basal ganglia and thalami will not evolve until approximately 2–3 days. Cortical and subcortical changes may even need to evolve over 5–7 days before a lesional pattern becomes apparent. In addition, CUS is used to rule out congenital malformation and hemorrhage before starting therapeutic hypothermia (Fig. 1).

### Perinatal arterial ischemic stroke

**Fig. 2:**
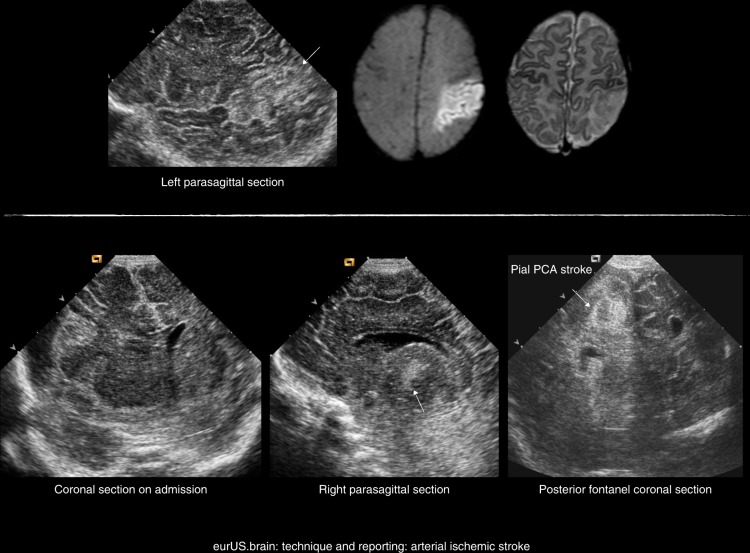
Technique and reporting: arterial ischemic stroke. Top: term infant with focal seizures on day 2: left posterior truncal MCA stroke; ultrasound and MRI (diffusion weighted and T2) on day 3. Bottom: vaginal breech delivery at 36 weeks’ GA, apnea, and tense fontanel at 24 h; pallor; and lowered consciousness: posterior cerebral artery stroke following uncal herniation due to right convexity subdural hematoma (left image on admission, other images on day 5) (arrow in the middle image: thalamic perforator stroke).

The gold standard to detect perinatal arterial ischemic stroke is MRI. Nevertheless, arterial stokes can often be detected with careful serial CUS imaging.^37^ An increased parenchymal echogenicity that becomes more apparent the first days after the insult and an abnormal perfusion pattern (restricted or luxury perfusion) can be detected in the vascular territory involved. In case of a persisted occlusion, Doppler imaging of the affected vessel can be informative (Fig. 2).

## Optimizing scan settings

**Fig. 3:**
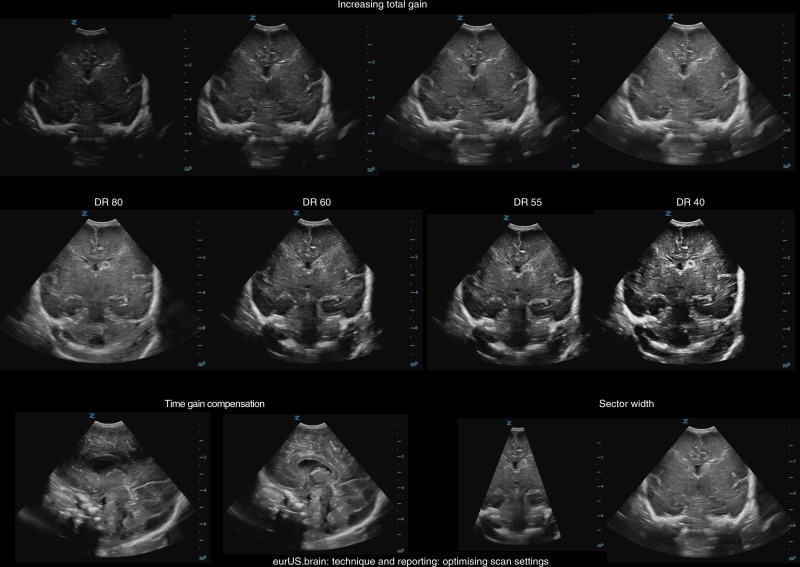
Technique and reporting: optimizing scan settings. Top row: gradually increasing total gain; middle row: gradually decreasing dynamic range settings; bottom row, left: correction of wrong time gain compensation setting at 2 cm depth; bottom row, right: different sector widths.

US systems have many adjustable settings, the configuration of which can dramatically affect image quality (Fig. 3).

Although neonatal CUS settings can be pre-programmed (which is recommended as starting point), settings will have to be optimized individually to prevent overlooking important features.^25^ Different pathologies often require specific settings. Knowledge of several aspects regarding hardware and software is essential for optimal use of the technique.

Table 2 provides an overview of adjustable settings. Besides operators’ skills, knowledge on the normal and abnormal developmental neuroanatomy (Table 3) and neonatal brain pathology (including time course of brain injuries) is important.^25^

**Table 2:** Optimizing scan settings.^38,39^

Depth	The depth control changes the maximum scanning range on screen. The depth range button usually changes the displayed image field in 1-cm gradation increments. Increasing depth means a reduction of the image resolution (the signal needs to cover a longer distance), therefore the frame rate and the resolution are both lower. The optimal depth depends on beam penetration and therefore on transducer frequency
Dynamic range (DR)	The DR controls the range of shades of gray displayed on the screen to be increased or decreased. It can make an image look either very black and white or very gray. It can remove low-level echoes and result in an image with more contrast
Focus point(s)	The focus (point) determines the depth at which the ultrasound beam is focused and creates the best possible lateral resolution at that depth. It is often marked by an arrow on the display. The focal zone is ideally positioned at (or just below) the object the operator wants to study. More than one “focal zone” can be selected, but this can slow down the image frame rate and can induce artefacts
Frequency	Adjusting the frequency allows the operator to improve the image resolution. Frequency is the number of cycles of acoustic waves per second. The unit is the Hertz (Hz) and one cycle per second is equal to 1 Hz; 10^6^ cycles/second is equal to 1 MHz. Diagnostic ultrasound has a frequency of 2–20 MHz. One should consider using higher frequencies (10–20 MHz) when scanning superficial and low frequencies (5–10 MHz) when scanning deeper structures
Gain (overall gain control)	The (overall) gain control will adjust the overall brightness of the real-time (B-mode) ultrasound image. Overall gain control amplifies all returning signals by a constant factor regardless of the depth (in contrast to time gain control). It has a similar effect to increasing the power. Gain is commonly expressed in decibels (dB). If the gain is increased too much, the noise will also be amplified leading to poor image quality
Power	The power button regulates the “output power” to the transducer (the intensity of the ultrasound pulses). The operator can increase the amplitude of the electric signal to the transducer and make the returning echo signals brighter. The risk, however, is that the acoustic exposure of the patient increases
Pulse repetition frequency (PRF)	The PRF controls the rate (per second) at which pulses of sound are transmitted by the transducer
Time gain compensation (TGC)	Increasing TGC (i.e., by adjusting the TGC sliders) amplifies signals from deeper structures to compensate for attenuation causing the signals from deeper structures to be weaker than signals returning from more shallow structures. The goal of TGC is to make the entire image look evenly bright. TGC sliders are used to adjust the gain in specific areas of the image (near-, mid-, and far-field). The idea is to have lower gain in the near field and higher gain deeper in the image where image quality is weaker. Most manufacturers offer a software feature that automatically optimizes the gain and overall contrast of the image. This feature analyses the tissue in the image and attempts to provide you with the most optimized image, but correction by the operator remains necessary

**Table 3:** Developmental neuroanatomy per gestational week.

12 weeks	Emergence of the insular cleavage Recognizable genu and splenium of corpus callosum Formation of primary cerebellar fissure
16 weeks	Completion of corpus callosum Recognizable cavum septum pellucidum Cerebellum covers fourth ventricle Formation of hippocampal gyrus Emergence of calcarine, parieto-occipital, and callosal sulci
20 weeks	Calcarine and parieto-occipital sulci formed Appearance of central sulcus and cingular sulcus Progressive thickening of corpus callosum (rostrocaudal direction)
24 weeks	Appearance of olfactory sulcus and of precentral and postcentral sulcus
28 weeks	Emergence of superior temporal sulcus and secondary sulci (parietal and temporal before frontal) Completion of circular sulcus of the insula (complete posterior opercularization around week 31)
32 weeks	Branching of precentral and postcentral sulci Appearance of midtemporal sulci Appearance of superior frontal sulcus

Patient safety should always be first priority; before scanning, the operator has to make sure the infant has stable vital signs. We recommend that the infant is supported by a parent or healthcare worker. Pressure of the probe has to be kept to a minimum and the gel should be warmed before. The probe should be small enough to fit in the AF. Often good-quality images can be made using a probe with a frequency of 7.5–11 MHz. Lower frequencies will allow better penetration improving visualization of deeper brain structures: the trade-off is loss of resolution. Loss of penetration depth using higher frequencies can partly be resolved by adapting the focus point or using multiple focus points. For standard CUS, the focus point is preferably the periventricular areas.

## Normal US anatomy in standard sections

### Anterior fontanel

Images are usually obtained through the AF. With optimal settings, this displays the supratentorial structures. Standard AF images are recorded in six coronal and five sagittal planes.^1,25^ In addition to standard planes, the whole brain is scanned to obtain an overview of its appearance. Any suspected lesion should be visualized in both planes. Routine Doppler visualization of large veins and arteries should be included.

#### Coronal planes

**Fig. 4:**
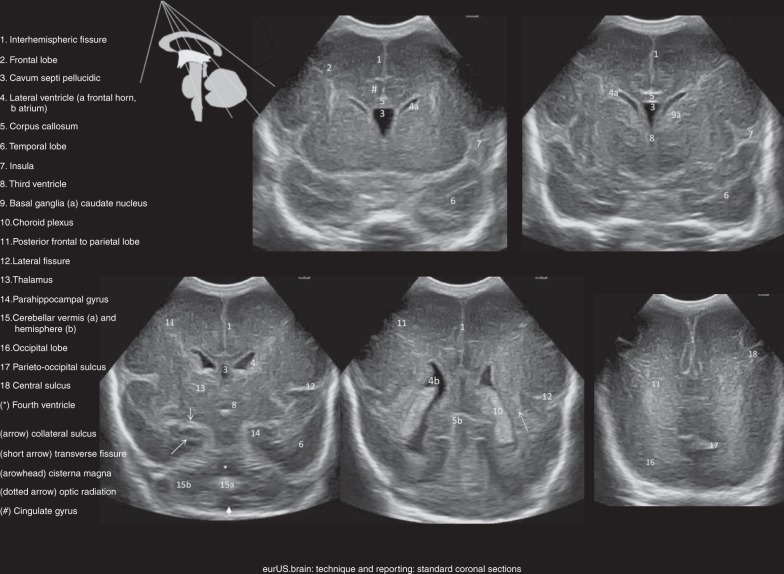
Technique and reporting: standard coronal sections. Standard coronal sections from anterior (top left) to posterior (bottom right); sectional planes in the scheme.

The transducer is placed in the middle of the AF such that the left half of the brain is displayed on the right-hand side of the monitor. The probe is angled forwards and backwards to scan the brain from the frontal lobes to the posterior parietal and occipital lobes. For reliable interpretation, it is crucial to obtain symmetrical images.^1,25^ Attention should be paid to both focal and bilateral abnormalities of cortex, white matter, deep gray matter, and ventricles. Doppler can be used not only to visualize the basilar artery, both internal carotid arteries, the middle (including perforators), and anterior cerebral arteries but also for assessing major venous drainage (i.e., flow patency of the superior sagittal sinus, sigmoid sinus, and internal cerebral veins; Fig. 4).

#### Sagittal and parasagittal planes

**Fig. 5:**
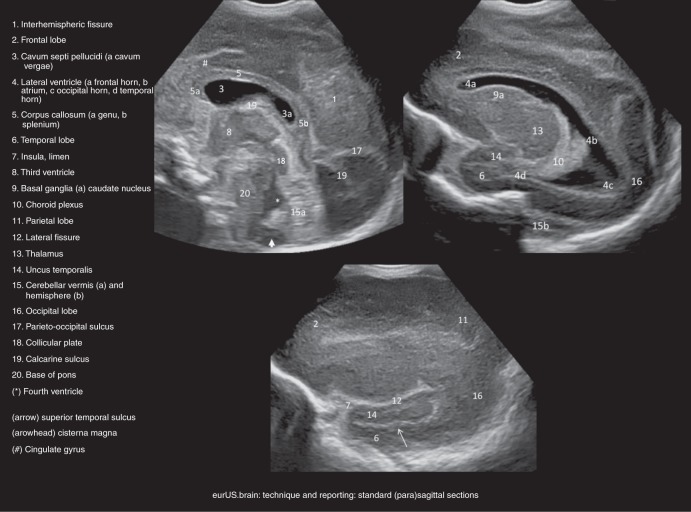
Technique and reporting: standard parasagittal sections. Sections from midline (top left) to insula (bottom).

For sagittal plane scanning, the transducer is rotated 90 degrees such that the anterior part of the brain is displayed on the left-hand side of the monitor. Images are obtained in the midsagittal plane and two parasagittal planes on each side. Regarding these parasagittal planes, it is important to mark the side of the brain that is visualized. The assessment of midline and near-midline structures includes: gyrus cinguli, corpus callosum, tela choroidea, third ventricle, cavum septi pellucidi (and Verga’s ventricle), cavum veli interpositi, cisterns, aqueduct, fourth ventricle, cerebellum, pons, and cisterna magna. The resistance index of the subcallosal anterior cerebral artery can be calculated. A value of >0.85 suggests a low diastolic flow and could indicate a steal phenomenon (i.e., persistent ductus arteriosus); a value <0.55 suggest a high diastolic flow (“luxury perfusion” in perinatal asphyxia). The parasagittal planes allow visualization of the lateral ventricles, the gangliothalamic “egg” (discerning thalamus, posterior limb of the internal capsule, globus pallidus, putamen, caudate nucleus) and uncus, fissure of Bichat, and hippocampi. With the use of three outward parasagittal planes, the insula can be inspected in detail: (1) opercular, (2) insular, and (3) fissural view (Fig. 5).

### Posterior fontanel

The posterior fontanel is located at the junction of the lambdoid and sagittal suture and is often large enough for insonation.^1,15^ This fontanel offers visualization of the occipital horns of the lateral ventricles, occipital lobes, and posterior fossa structures. Imaging through this fontanel improves the detection of limited IVH and lesions in the occipital lobes and better defines posterior fossa malformations.^14^ Furthermore, posterior fontanel views allow detecting posterior cerebral artery stroke and the effects of severe hypoglycemia.

**Fig. 6:**
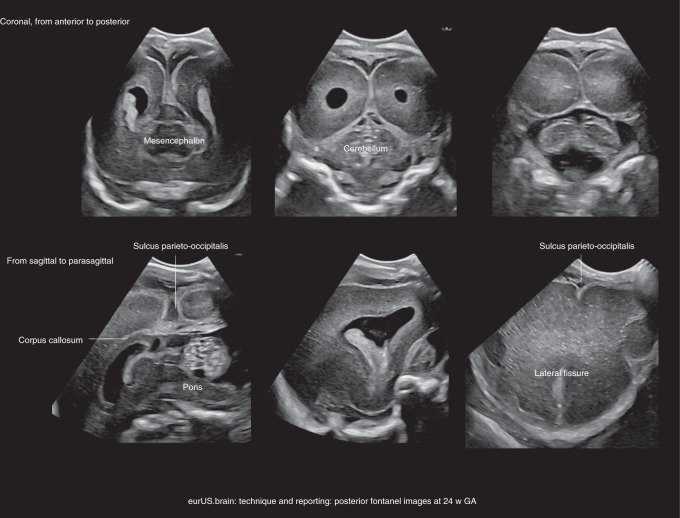
Technique and reporting: posterior fontanel images at 24 weeks’ GA. Posterior fontanel section, top coronal (left anterior to right posterior), bottom sagittal and parasagittal.

Posterior fontanel CUS includes both coronal and sagittal views. The infant is in supine position with the head turned to one side and slightly lifted to facilitate transducer movement (Fig. 6).

#### Coronal planes

The transducer is placed in the middle of the posterior fontanel such that the left half of the brain is displayed on the right-hand side of the monitor. The probe is angled from the most superior coronal plane with trigones and choroid plexus to the inferior coronal plane with occipital horns, tentorium, and infratentorial structures.

#### Sagittal and parasagittal planes

For images in sagittal planes, the transducer is turned 90 degrees. The superior part of the brain is displayed on the left-hand side of the monitor. Imaging starts with a midsagittal view followed by two parasagittal views on each side.

### Temporo-squamosal fontanel

**Fig. 7:**
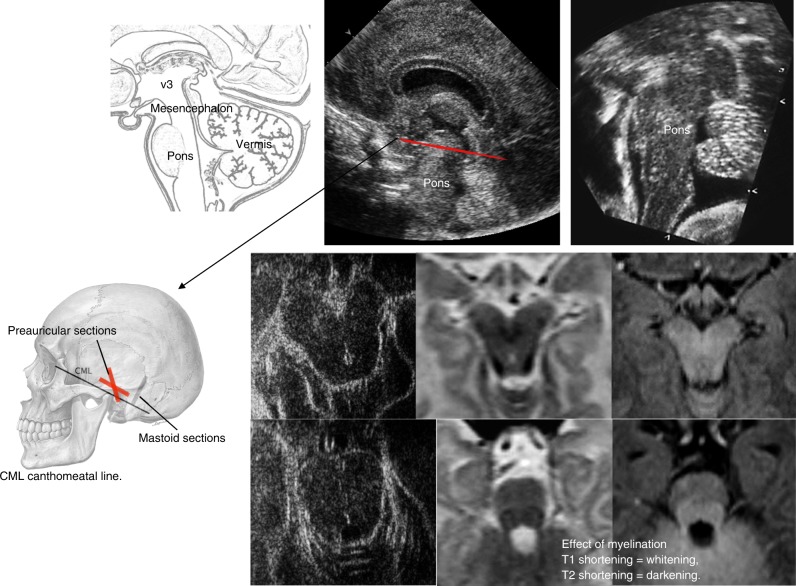
Technique and reporting: temporo-squamosal section. Top: sagittal section through brainstem and cerebellar vermis, compared with a sagittal scan of an infant of 27 weeks gestation, taken though the anterior fontanel with an 8.5 MHz scanhead; far right: sagittal 7.5 MHz ultrasound section of the area, taken through the posterior fontanel of an infant with cleidocranial dysplasia. Bottom: temporo-squamosal sections (parallel to the cantho-meatal line, indicated in red on the top scan): the echopoor mesencephalon looks like a butterfly; the cerebral peduncles and tectal lamina are surrounded by hyperechoic cisterns and parts of the tentorium; bright reflections in the posterior part of the brainstem coincide with the walls of the aqueduct; in the basal cisterns, the arteries of the circle of Willis show as short, pulsating lines; term MRI sections for comparison.

Transverse views of the brain stem are obtained through the temporal window. The infant is positioned with the head turned to one side. The transducer is placed in a horizontal position above and anterior to the external auditory meatus and then slightly adjusted until a view of the brain stem is obtained. Important anatomic structures to be observed are the thalami, midbrain, third ventricle, aqueduct of Sylvius, and the perimesencephalic cistern. Scanning through the temporal window allows detecting brain stem abnormalities and provides an overview of the ventricular system in cases of congenital or acquired hydrocephalus. It also allows Doppler flow measurements in the circle of Willis and visualization of cerebrospinal fluid flow in the aqueduct in some instances (Fig. 7).

### Mastoid fontanel

The mastoid fontanel is located behind the ear at the junction of the temporal, occipital, and posterior parietal bones.^1,15,18^ The use of this fontanel improves visualization of the posterior fossa (see related paper in this issue). This results in a better detection of both congenital and acquired posterior fossa abnormalities and in particular of cerebellar hemorrhage in preterm infants.^17,19,20,26–28^ The infant is positioned with the head turned to one side. The transducer is placed behind the helix of the ear and then slightly moved until reproducible views are obtained. Imaging is performed in both transverse (axial) and coronal planes. An abnormality or a suspected abnormality can be confirmed by also scanning the opposite side. Both the transverse and sigmoid sinuses can be clearly visualized using the mastoid fontanel window. In many infants, reliable transverse cerebellar diameters can be measured after visualization of both cerebellar hemispheres.

#### Transverse (axial) planes

For transverse views, the transducer is placed in a horizontal position, almost parallel to the orbitomeatal line.^13,27^ Superior transverse views show the superior vermis, cerebral peduncles, aqueduct, and perimesencephalic and quadrigeminal cisterns. Middle transverse views include the vermis and hemispheres at the level of the fourth ventricle, the pons, and parts of the temporal lobes. Inferior axial views show inferior parts of the cerebellar hemispheres, vermis, and cisterna magna.

#### Coronal planes

Coronal views are obtained with the transducer placed along the coronal suture.^13,27^ Anterior coronal views show the pons, tentorium, fourth ventricle, cerebellar vermis, hemispheres, and the cisterna magna. Posterior coronal views show posterior parts of the lateral ventricles, cerebellar vermis, hemispheres, and cisterna magna.

### Foramen magnum

The use of the foramen magnum is not ideal for scanning the inferior posterior fossa and cranio-cervical junction, but this foramen can serve as an additional window to assess the anatomy in these areas and to better define pathology, for example, in posterior fossa malformation (e.g., Chiari malformation) and post-hemorrhagic hydrocephalus.^16,29^ The infant is placed in a lateral position with the head slightly flexed forward, similar to positioning for a lumbar puncture. Images can be obtained in both sagittal and transverse planes, as described by Brennan et al.^16^

## Reporting

Clear, concise, and timely reporting of the CUS findings and prudent interpretation are important. Abbreviations should be avoided. Reporting should include both normal and abnormal findings. Specific diagnoses and/or differential diagnoses should be stated, keeping in mind that ultrasound is a surrogate of neuropathology, reflected in speckle clusters of variable organization and brightness. The extent of lesions should be detailed using appropriate anatomical terminology. If grading systems are used, these should be specified (e.g., IVH grade II).^38–40^ Variations from normal size should be documented by—preferably standardized—measurements. Regarding the lateral ventricle size, good intraobserver and interobserver agreement have been documented for the measurement of the anterior horn width and the thalamo-occipital distance and calculating the ventricular index according to Levene.^40^ In some cases, it may be helpful to compare findings to those of previous exams, so as to get an impression of the evolution of lesions and guide intervention (e.g., cerebrospinal fluid drainage in infants with post-hemorrhagic ventricular dilatation). A recommendation for follow-up imaging or further investigation should be added where appropriate. Images of all standard sections should be recorded and documented—preferably in a format that permits later measurements (e.g., DICOM files). Additional views should be added whenever needed, including video sequences. Limitations that influence the quality of the CUS examination (e.g., small fontanel, limited examination time because of the infant’s unstable condition, or technical problems) should be mentioned.

## Safety of CUS

CUS of the newborn brain is considered non-invasive. Nevertheless, acoustic waves can cause thermal and mechanical effects in the interrogated tissue. Significant heating of the brain has been observed in neonatal animal models.^41^ It is unknown whether heating of the brain affect neuronal integrity, but it seems unlikely that this would occur in routine practice. State-of-the-art CUS devices monitor and display the thermal index and mechanical index. The thermal index is the ratio of acoustic power to the power needed to raise the temperature by 1 °C in the tissue being examined.^38,39^ The mechanical index represents the probability of cavitation, which is the formation of bubbles. It is calculated as peak rarefactional pressure divided by the square root of the US frequency.^38,39^ The number of studies on adverse effects of US on the developing brain with state-of-the-art US devices—especially in the most immature premature infants, those born before 28 weeks gestation—are still very limited.^41^ Therefore, clinicians would do well to weigh risk against benefit. Users of US devices should be aware about the biophysical mechanisms and how US settings effect those. In the absence of sufficient safety data, apply the ALARA principle (“as-low-as-reasonably-achievable”). Furthermore, qualified technical staff should be present to service the equipment. The US probes are made of vulnerable components and must be handled with care. Damage to probe housing can lead to electric current leaks. Finally, the operator should consider patient safety at all times: handling critically ill newborns can be riskful, e.g., when pressure is applied to the fontanel. The probes should be thoroughly cleaned, following the manufacturer’s instructions.

## Future perspectives

Technological progress has led to an exponential increase in the computational speed of US systems and the introduction of new applications. “Ultrafast US” systems have been developed that make use of plane waves and multi-core central processing units and can process thousands of frames per second.^42–44^ These systems have revolutionized the temporal resolution and sensitivity. Quantitative mapping of cerebral vascular dynamics has been made possible by high-frequency imaging. Demené et al. used ultrafast Doppler to map the vasculature dynamics of the neonatal brain in vivo.^43^ Within a single cardiac cycle, simultaneous estimations of full Doppler spectra (in all pixels) could be obtained. Tanter et al. used ultrafast imaging to make “functional” US images of the rat brain.^45^ The rat whiskers were stimulated and this technique (similar to fMRI) was used to quantify cerebral blood perfusion differences based on vasodilatation in active parts of the brain. Demené et al. mounted a similar light-weight US probe (combined with electroencephalogram electrodes) on the head of newborns to study their cerebral perfusion during quiet sleep and active sleep.^42^ Other new developments that are expected to improve diagnostic US of neonatal brains include: shear wave imaging, contrast enhanced imaging, advanced three-dimensional imaging, and image registration. Again, it is important to always be wary of safety concerns when powerful new diagnostic equipment is used in neonatal clinical studies.^41^
